# The Legacy of James Marion Sims: History Revisited

**DOI:** 10.7759/cureus.69484

**Published:** 2024-09-15

**Authors:** Priyal Shende, Akshay Jagtap, Bansari Goswami

**Affiliations:** 1 Obstetrics and Gynecology, Dr. D.Y. Patil Medical College, Hospital and Research Centre, Dr. D. Y. Patil Vidyapeeth (Deemed to be University), Pune, IND

**Keywords:** enslaved people, modern gynaecology, society, surgical technique, vesicovaginal fistula

## Abstract

The title "Father of Modern Gynecology" is often attributed to Dr James Marion Sims, a pioneering American physician whose contributions to gynecology have profoundly influenced modern medical practice. Born in 1813, Sims developed several innovative surgical techniques and instruments that revolutionized the treatment of gynecological conditions. Among his most notable contributions is the Sims speculum, which remains a fundamental tool in gynecological examinations today. Sims is also credited with pioneering surgical techniques for repairing VVFs, previously deemed untreatable. His work, primarily conducted in the mid-19th century, laid the foundation for modern gynecological surgery and significantly advanced women's healthcare. However, Sims' legacy is also marked by ethical controversy. His early research involved experimental surgeries on enslaved African-American women, conducted without the use of anesthesia. These practices have sparked critical discussions regarding medical ethics, informed consent, and the historical exploitation of marginalized groups under the guise of scientific progress.

This review article explores the dual facets of James Marion Sims' legacy, acknowledging his crucial role in shaping modern gynecology while critically examining the ethical implications of his methods. Such a discussion is vital for understanding the evolution of medical practices and the ongoing need for stringent ethical standards in clinical research and patient care.

## Introduction and background

**Figure 1:**
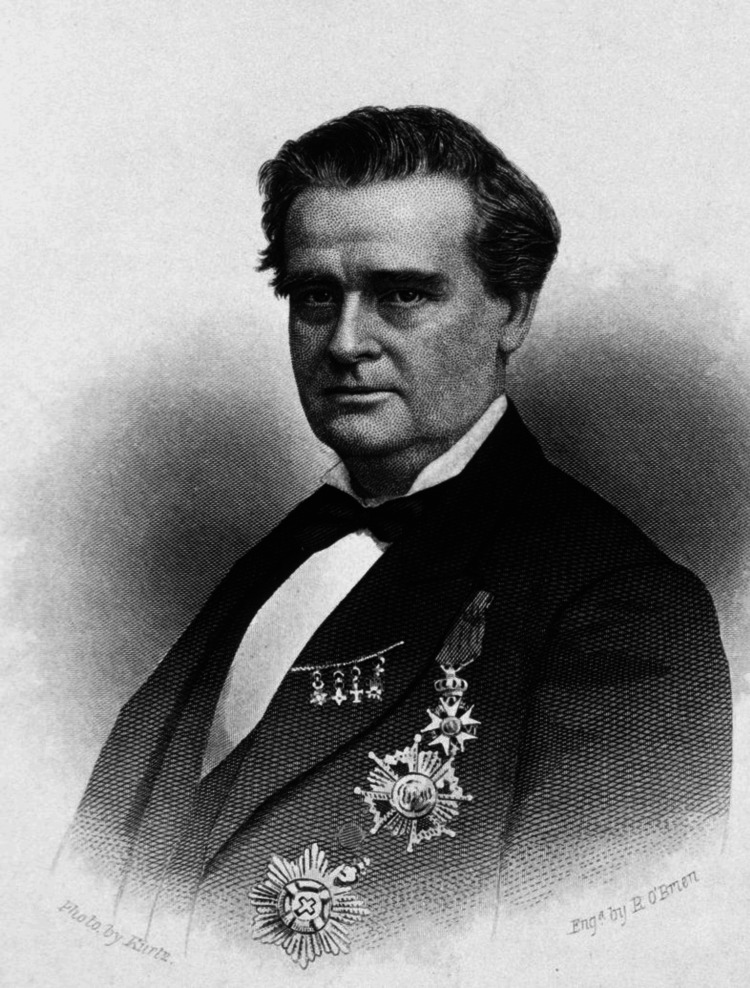
J. Marion Sims Source: [2].

James Marion Sims (January 25, 1813 South Carolina- U.S. - November 13, 1883) (Figure 1) is often hailed as the "Father of Modern Gynecology" due to his groundbreaking contributions to the field, particularly during the mid-19th century. He earned this title through several key achievements, including the development of a successful surgical technique for repairing vesicovaginal fistulas (VVFs), a condition that was once considered untreatable and caused significant suffering among women after childbirth. Sims’ innovative approach provided a reliable solution, greatly improving the lives of affected women and establishing a foundation for future gynecological surgeries. Additionally, he invented the Sims speculum, an instrument that enhanced gynecological examinations and continues to be a standard tool in the field today. In 1855, Sims founded the New York Woman’s Hospital, the first institution dedicated exclusively to women’s health, which became a leading center for gynecological care and education, further solidifying his influence. Sims also significantly contributed to medical literature, publishing numerous papers and books that advanced surgical techniques and the overall body of knowledge in gynecology. As an educator and mentor, he played a crucial role in shaping the future of the profession, teaching many students who would go on to become influential physicians themselves. At a time when women’s health issues were often overlooked, Sims focused on conditions primarily affecting women, advocating for specialized care and helping to establish gynecology as a distinct and essential medical discipline. James Marion Sims was one of the most renowned and revered physicians in the United States, with his fame extending even to Europe. In 1876, he was elected President of the American Medical Association, and he proudly claimed to be the second-wealthiest doctor in the country [1,2].

## Review

Roots in the market for enslaved people

James Marion Sims, born in 1813 in Lancaster County, South Carolina, pursued a medical career during a period when the educational and training requirements for doctors were far less stringent than they are today. After completing a brief internship, followed by a three-month medical course and a year of study at Jefferson Medical College, Sims began practising medicine in Lancaster. However, following the deaths of his first two patients, he moved to Montgomery, Alabama, seeking a fresh start [3].

Sims gained recognition among wealthy white plantation owners in Montgomery for treating their enslaved laborers to medical attention. Professor of medical humanities at George Washington University Vanessa Gamble points out that Sims' medical practice was closely linked to the slave trade. In the center of Montgomery's trading district, he founded an eight-bed hospital where he provided care for enslaved people who were brought in from plantations with more serious medical issues. Because their ability to labor and procreate was often considered the only thing that made these enslaved valuable, Sims "patch them up" in order to keep them working for their owners.

The idea of "soundness" was pivotal in this practice, representing an enslaved person's ability to labour and bear children. Women suffering from conditions like VVFs, which compromised their reproductive functions, were deemed "unsound" and thus less valuable to their owners [4].

Sims originally had little interest in treating female patients, which was common among 19th-century doctors, many of whom regarded gynecological work as unpleasant and inappropriate. However when he was asked to help a woman who had fallen off a horse and was experiencing back and pelvic discomfort, his perspective changed. Sims understood he had to check her vagina directly in order to treat her properly. Leaning forward, he put her on all fours and used his fingers to improve his vision.

This led to the invention of a device that would eventually evolve into the modern speculum, which he initially created by bending the handle of a pewter spoon. This experience marked the start of Sims' dedication to gynecology and his later contributions to the field [5]. In defense of Sims, others contend that, as a Southern slaveholder, he was a product of his day and that, in this case, the treatment of enslaved women with fistulas was probably something they desired enough to consent to. However these women's viewpoints were not recorded, and at the time, the only legally required consent came from their proprietors, who had a financial stake in their recovery [4].

Contributions of James Marion Sims

Vaginal Surgery

Before Sims' work, VVFs were a poorly understood and often untreated condition. In the early 19th century, Sims developed a surgical approach to repair VVFs. His technique involved the use of a specialized instrument called the Sims speculum, which allowed better visualization of the vaginal canal and the affected area. He used sutures to close the fistula and restore the normal anatomy of the bladder and vagina. Sims' method became a standard treatment for VVFs and significantly improved the quality of life for women suffering from this condition.

Instrumentation

Sims invented several medical instruments, including the Sims speculum and the Sims sigmoid catheter, which became standard tools in gynecology. However, there has been a petition campaign to remove Sims name from these instruments, such as the Sims uterine curette, uterine sound, vaginal retractor, uterine scissors, and rectal speculum. The proposal suggests that new names be chosen by a committee of People of Color (POC). The Sims speculum significantly improved the ability of physicians to examine the cervix and vagina, facilitating more accurate diagnoses and safer surgical procedures. Although it has been modified over time, the basic design of the Sims speculum is still in use today.

Examination and Surgical Positioning

Sims introduced a new patient position for gynecological procedures, now known as the "Sims' position." In this position, the patient lies on their left side with their right knee drawn up toward the chest. The left arm is placed behind the back, and the head is slightly turned to the side. This position provides better access to the vagina and cervix than the traditional lithotomy position (where the patient lies on their back with legs raised and supported). It is particularly useful for certain types of gynecological surgeries and for examining patients who cannot tolerate the lithotomy position [3,4].

Establishment of the New York Woman’s Hospital

At the time, medical institutions dedicated specifically to women's health were almost non-existent. Sims recognized the need for a specialized hospital to address the unique medical needs of women. In 1855, Sims founded the New York Woman’s Hospital, the first hospital in the world dedicated exclusively to the treatment of woman’s diseases. The hospital became a leading center for gynecological research, training, and care. Under Sims’ direction, the hospital advanced the study and treatment of various gynecological conditions. It also provided a training ground for future gynecologists, helping to professionalize and standardize the field of gynecology.

Fertility Treatment

Sims contributed to fertility treatment through techniques such as artificial insemination and the postcoital test, which evaluates sperm presence and movement after intercourse.

Cancer Care

Contrary to the prevailing belief at the time that cancer was contagious, Sims argued for the admission of cancer patients to the Woman's Hospital, helping to advance the care and understanding of the disease.

Groundbreaking Work in Gynecological Surgery

Sims promoted the use of laparotomies to drain wounds, mend internal damage, and halt bleeding from bullet wounds in the abdomen. When President James Garfield was the target of an assassination attempt, his knowledge was requested, and he responded by telegram from Paris. Sims' suggestions were finally adopted by the medical community. Sims created methods for the treatment of uterine prolapse and cervical carcinoma in addition to fistula repair.

Gallbladder Surgery

In 1878, Sims performed surgery to drain a distended gallbladder and remove its stones. Although he believed this was the first such operation, a similar case had been reported in Indianapolis in 1867. Nonetheless, Sims' work in this area was significant and added to the body of surgical knowledge [3].

Ethical considerations and criticism

James Marion Sims was celebrated as a pioneering surgeon during his lifetime, and his contributions were widely respected even after his death. However, his reputation suffered a significant decline in the mid-20th century due to intense scrutiny of his ethical practices. The primary source of this criticism is his use of enslaved African American women as subjects for experimentation while attempting to cure VVFs [5-7]. From late 1845 to 1849, Sims conducted numerous surgeries on these women in a small hospital behind his Montgomery, Alabama home, including 30 operations on Anarcha, who endured severe fistulas. Contemporary critics, such as Durrenda Ojanuga in the Journal of Medical Ethics, harshly condemn Sims for exploiting vulnerable Black women, portraying him as a cruel individual who performed unnecessary surgeries without consent for personal gain. However, this paper argues that such critiques may not fully align with historical records and overlook the severe clinical issues faced by these women, reflecting Herbert Butterfield's notion that focusing on partial truths can lead to distorted historical interpretations [8,9].

Modern critics of James Marion Sims primarily make three allegations about his work with VVFs: first, experimenting on enslaved individuals was unethical due to lack of informed consent; second, his use of anesthesia only for white patients suggests racial discrimination; and third, other physicians achieved similar medical advances ethically with informed consent from white women. However, a review of primary sources indicates that these claims may be based on flawed assumptions and inadequate historical research, overlooking the severe clinical challenges of treating VVFs in the 19th century [7-11].

Critics like Ojanuga argue that medical experimentation on enslaved individuals was inherently unethical, but they often overlook the distinction between non-therapeutic and therapeutic experiments. Non-therapeutic experiments offer no direct benefit to participants, whereas therapeutic experiments aim to provide significant medical relief. When Sims began his work on VVFs, no effective treatments existed, and patients faced ongoing suffering [12,13]. French surgeon Alfred Velpeau noted in 1847 that no successful cures had been widely accepted, with many surgeons experiencing repeated failures. Given their dire circumstances, it is understandable that these women, despite being enslaved, would choose to undergo surgery for a chance at relief. This willingness to try new treatments was a common response among patients suffering from such conditions both then and now [14,15]. Critics like Vanessa Northington Gamble have accused James Marion Sims of "anesthetic racism," suggesting that he used anesthesia only for white patients. However, Sims conducted his early surgeries before ether anesthesia was discovered on October 16, 1846, nearly a year after he began operating on enslaved individuals. At the time of his early work, anesthesia was not yet available, and its use was not widespread even after its discovery. Thus, claims that Sims intentionally withheld anesthesia from enslaved patients are inaccurate given the historical context [16].

Ojanuga's claim that white women could not endure even a single operation by Sims due to intense pain is inaccurate. In 1849, Sims documented successfully performing three surgeries without anesthesia on a white woman with a fistula. Terri Kapsalis suggests that anesthetics allowed Sims to treat white women and establish the Woman's Hospital in New York. However, Sims himself stated in 1857 that he avoided anesthesia for fistula surgeries, believing the pain was not severe enough to warrant its risks. This reflects poor clinical judgment rather than "anesthetic racism," as anesthesia was still experimental at the time [17].

In June 1849, J. Marion Sims experimented with silver sutures to repair fistulas after suspecting that silk sutures caused infections. This new approach, used on Anarcha who had previously undergone 12 failed surgeries, showed no inflammation and led to successful healing. Sims claimed to have cured Anarcha and other patients, publishing his refined technique in 1852 [18]. Although Sims is a recognized pioneer in gynecological surgery, his ethical practices remain controversial. While human experimentation in the 19th century was not unique to Sims, such as Ephraim McDowell's surgeries on enslaved women and Werner Henle's questionable experiments in the 20th century, Sims' work led to significant advancements without reported fatalities. The varying levels of criticism for historical figures like Sims, McDowell, and Henle highlight complexities in assessing medical ethics and contributions [19,20].

The civil conflict and post-conflict victory

In 1861, J. Marion Sims left the U.S. and spent about seven years in Europe, where many medical professionals became interested in his innovative surgical techniques and began replicating them. He gave demonstrations in cities like Edinburgh, Dublin, London, and Paris. During this time, his focus shifted to treating wealthier patients and addressing infertility, motivated by concerns about declining birth rates among the elite. Sims claimed he could earn approximately $50,000 annually in Europe but returned to New York in 1868 [21].

Upon his return, despite facing some resistance, particularly from female administrators known as "Lady Supervisors," Sims successfully re-established his practice and expanded the woman’s hospital. His financial status improved significantly during the Reconstruction era, and he considered himself the second wealthiest physician in America. His Southern origins, Confederate connections, and racial views did not impede his success, particularly among Europe's colonial elite. Sims received numerous accolades, including Germany’s Iron Cross, France’s Legion of Honor, and recognition from Italy, Spain, and Portugal. He was also honored with memberships in medical societies across several European cities, including Edinburgh, Brussels, Berlin, Paris, and Dublin.

Sims is commemorated in the U.S. with monuments such as a statue in Central Park. The plaque at the statehouse grounds in Columbia, South Carolina, reads:

"Where there is love for humanity, there is also love for art. He founded the science of gynecology, was honored worldwide, and passed away with the blessing of humanity. The foremost surgeon of his time in service to women, caring for both empresses and slaves alike [22]."

Sims' achievements were highly esteemed by the medical community and documented in global journals. His international success and recognition after the Civil War highlight how medical professionals continued to uphold racial and gender biases while advancing their fields. At Sims' memorial service in 1883, a physician expressed pride, noting:

"I cannot help but feel immense pride that this great and noble man was born in the South. Despite being far from major centers of medical knowledge, this region can take pride in producing the bold and pioneering gynecologist J. Marion Sims, whose brilliance, skill, and determination transformed gynecology into a science [23]."

## Conclusions

Evaluating the medical ethics of historical figures can be challenging when considered through the lens of contemporary values, which address issues of race, gender and class differently than was the case in the past. J. Marion Sims was a committed physician who practiced in a slaveholding society and often treated enslaved individuals with genuine medical needs. Among the severe issues faced by many 19th-century women, both white and Black, was VVFs from childbirth. Sims’s surgeries between 1845 and 1849 aimed to address this debilitating condition, which was considered untreatable at the time. Though his initial efforts were unsuccessful, they were performed with therapeutic intent and, according to available records, with the patient's consent and cooperation. At that time, there were no effective treatments besides surgery, and the suffering caused by these injuries was widely acknowledged as unbearable. While it is true that enslaved people were often subjected to significant abuse, arguing that no attempts should have been made to alleviate their suffering, especially when no other options existed, seems ethically problematic. Despite his other faults, Sims pursued his goal with determination, and his work ultimately benefited many patients both then and in the future.

## References

[1] (2024). The ‘father of modern gynecology’ performed shocking experiments on enslaved women. https://www.history.com/news/the-father-of-modern-gynecology-performed-shocking-experiments-on-slaves.

[2] (2024). File:James Marion Sims. https://commons.wikimedia.org/wiki/File:James_Marion_Sims.jpg.

[3] (2024). James marion sims: Father of modern gynecology or abuser?. https://cpp-college.netlify.app/programs/education-blog/james-marion-sims-father-modern-gynecology-or-abuser.

[4] Barker-Benfield GJ (1999). The Horrors of the Half-Known Life. https://www.routledge.com/The-Horrors-of-the-Half-Known-Life-Male-Attitudes-Toward-Women-and-Sexuality-in-19th-Century-America/Barker-Benfield/p/book/9780415925006?srsltid=AfmBOoqs3zMspOJw6jOTrfMlkbgfiURM7MBZJWp1miQMeOs5NqixSlN0.

[5] Gamble VN (1997). Under the shadow of Tuskegee: African Americans and health care. Am J Public Health.

[6] Byrd WM, Clayton LA (1992). An American health dilemma: a history of blacks in the health system. J Natl Med Assoc.

[7] Haseltine Haseltine, Florence P (1997). Public privates: performing gynecology from both ends of the speculum. New Engl J Med.

[8] Ojanuga D (1993). The medical ethics of the 'father of gynaecology', Dr J Marion Sims. J Med Ethics.

[9] (2024). The whig interpretation of history. http://seas3.elte.hu/coursematerial/LojkoMiklos/Butterfield,_The_Whig_Interpretation_of_History_highlights.pdf.

[10] Axelsen DE (1985). Women as victims of medical experimentation: J. Marion Sims' surgery on slave women, 1845-1850. Sage.

[11] Weisz G (2000). From midwives to medicine: the birth of American gynecology. Med Hist.

[12] (1847). New elements of operative surgery. Buffalo Med J Mon Rev Med Surg Sci.

[13] (1844). A Treatise on Operative Surgery. Philadelphia: Carey & Hart. https://www.christies.com/en/lot/lot-4414399.

[14] Rajaian S, Pragatheeswarane M, Panda A (2019). Vesicovaginal fistula: review and recent trends. Indian J Urol.

[15] Wall LL, Arrowsmith SD, Briggs ND, Browning A, Lassey A (2005). The obstetric vesicovaginal fistula in the developing world. Obstet Gynecol Surv.

[16] Chaturvedi R, Gogna RL Retd (2011). Ether day: an intriguing history. Med J Armed Forces India.

[17] (1858). Silver sutures in surgery. The anniversary discourse before the New York Academey of Medicine. Br Foreign Med Chir Rev.

[18] Moir C (1940). J. Marion Sims and the vesico-vaginal fistula: then and now. Br Med J.

[19] de Costa CM (2003). James Marion Sims: some speculations and a new position. Med J Aust.

[20] (2017). This American Doctor Pioneered Abdominal Surgery by Operating on Enslaved Women. https://www.smithsonianmag.com/history/father-abdominal-surgery-practiced-enslaved-women-180967589/.

[21] (2024). Trial and Error: J. Marion Sims and the Birth of Modern Gynecology in the American South. https://cpb-us-e1.wpmucdn.com/sites.ucsc.edu/dist/f/482/files/2017/08/SocialBiog.Engineer.pdf.

[22] Kaiser IH (1978). Reappraisals of J. Marion Sims. Am J Obstet Gynecol.

[23] (1950). Woman's surgeon: the life story of J. Marion Sims. JAMA.

